# Association Study between the Sentinel Lymph Node Biopsy and the Clinicopathological Features of Patients with Cervical Cancer

**DOI:** 10.1155/2022/9697629

**Published:** 2022-08-26

**Authors:** Gaoli Niu, Yajuan Ren, Yanhong Zhai

**Affiliations:** Department of Gynecologic Oncology, The First Affiliated Hospital of Henan Polytechnic University (The Second People's Hospital of Jiaozuo), No. 17 Minzhu South Road, Liberated Area, Jiaozuo, Henan 454000, China

## Abstract

**Objective:**

The incidence of cervical cancer is increasing year by year, which seriously threatens the health of female patients. This study is aimed at investigating the association of sentinel lymph node biopsy (SLNB) with clinicopathological features in cervical cancer patients.

**Methods:**

Patients diagnosed with cervical cancer in our hospital from February 1, 2019, to June 30, 2021, were selected as the research subjects. Statistical analysis was performed on the SLN examination of patients with cervical cancer with different pathological characteristics and the correlation between the positive rate of SLN detection and the pathological characteristics of cervical cancer.

**Results:**

A total of 59 patients with cervical cancer were included in this study, the SLNB detection rate was 94.92%, 15 patients had lymph node metastasis, and the metastasis rate was 25.42% confirmed by histopathology. Thirteen of them had SLN metastases, and the other 2 had non-SLN metastases. The sensitivity of SLNB was 86.67%, and the false negative rate was 13.33%. Statistical analysis results showed that there was no significant difference in the positive rate of SLN among cervical cancer patients with different FIGO stages, pathological types, degree of differentiation, depth of invasion, and tumor size. In addition, the results of Pearson's correlation analysis showed that the positive rate of SLN was not significantly correlated with the FIGO stage, pathological type, degree of differentiation, depth of invasion, and tumor size of cervical cancer.

**Conclusion:**

SLNB has a high sensitivity, safety, and feasibility in the diagnosis and evaluation of lymph node metastasis in cervical cancer. There is no significant correlation between SLNB and the clinicopathological features of cervical cancer.

## 1. Introduction

Cervical cancer is a malignant tumor of the female reproductive system. Carcinoma in situ is more common in women aged 30-35 years, and infiltrating carcinoma is more common in women aged 45-55 years. In recent years, the incidence of cervical cancer continues to increase, especially in younger patients, which poses a great threat to the quality of life and physical and mental state [1, 2]. Lymph node metastasis is an important factor affecting the prognosis of cervical cancer. However, the rate of pelvic lymph node metastasis in patients with early cervical cancer is only about 10%-34%, and 2/3 of patients without lymph node metastasis choose to undergo unnecessary pelvic lymph node resection, which may damage the body's immune system. In addition, some patients have clinical symptoms such as perineal edema, bipedal edema, and lymphocyst [3–5]. Therefore, early detection of lymph node metastasis is significant to avoid unnecessary trauma from lymph node dissection and to preserve fertility [6].

SLN is the first lymph node that receives lymphatic drainage in the tumor area, which can inhibit the proliferation of tumor cells through the lymphatic channel. Therefore, it is believed that SLNB is useful in the evaluation of pelvic lymph node metastasis [7]. Currently, the clinical value of SLNB in predicting lymph node metastasis in breast cancer and other malignant tumors has been confirmed. However, due to the location of the cervix, the lymphatic drainage of cervical cancer is more complicated, and the accuracy of using SLNB to determine the lymph node metastasis of cervical cancer in related studies is not clear [8].

This study is aimed at investigating the correlation between SLNB and the clinicopathological features of patients with cervical cancer.

## 2. Materials and Methods

### 2.1. Selection Criteria

The patients with cervical cancer in our hospital from February 1, 2019, to June 30, 2021, were selected. The inclusion criteria for this study were as follows: (1) cervical cancer was diagnosed according to the diagnostic criteria [9] and confirmed by pathological examination; (2) International Federation of Gynecology and Obstetrics (FIGO) stage I-IIb; and (3) MRI (no suspicious lymph nodes were found in the pelvis or abdominal cavity on MRI) or computed tomography (CT). The exclusion criteria were as follows: (1) patients with benign and malignant tumors; (2) with a history of pelvic surgery, surgical contraindications, or other gynecological diseases; and (3) received chemoradiotherapy before study enrollment. All patients signed informed consent, and this study was approved by the ethics committee of our hospital.

### 2.2. SLNB Procedure

One day before the surgery, 0.4 mL dextran labeled with 37 MBq Technetium-99m (99mTc) was injected into the submucosa of the cervix at approximately the 2 and 10 o'clock positions. The syringe (1 mL) with an intracardiac injection needle was injected at the edge of the tumor approximately 0.5 cm into the submucosa. After the injection, SLN radionuclide imaging was performed at 15 min, 30 min, 60 min, and 120 min, respectively. Subsequently, an extensive hysterectomy/lymph node dissection was performed under general anesthesia. After the surgery, the isolated lymph node specimens were detected using a *γ*-detector and compared with the adipose tissue surrounding the ipsilateral lymph node. If the count increased by at least ten times, the presence of an SLN was considered, and the lymph node was separated. The SLN and NSLN specimens were subjected to pathological examination, and the results were recorded.

### 2.3. Observation Indicators

The SLN examination of the patients in this group was statistically analyzed. The SLNB evaluation criteria were as follows: SLN and NSLN had metastasis to negative, SLN had metastasis to positive, SLN had not metastasis, and NSLN had metastasis to false-negative. Sensitivity was calculated as the SLN metastasis number/lymph node metastasis number ×100%. The false-negative rate was calculated using the following formula: false − negative number/lymph node metastasis number × 100%. The SLN examination of patients with cervical cancer with different pathological characteristics (FIGO staging, pathological type, differentiation degree, infiltration depth, and tumor size) was statistically analyzed. The correlation between the positive rate of SLN detection and pathological characteristics of cervical cancer was statistically analyzed.

### 2.4. Statistical Analyses

All statistical analyses were performed using the SPSS version 22.0 (IBM SPSS statistics, USA). The *t*-test was used to analyze the measurement data. The chi-square test was used to analyze count data (numbers and percentages). In addition, Pearson's correlation analysis was used to determine the correlation between the SLN positivity rate and the pathological features of cervical cancer. The two-sided *P* less than 0.05 was set as statistical.

## 3. Results

### 3.1. Baseline Characteristics

The study initially included 60 patients, but 1 patient who had received chemotherapy prior to the study was excluded. A total of 59 patients with cervical cancer, aged 33-64 years (mean 48.64 ± 12.91 years), were included. According to FIGO staging, there were 19 cases in stage I, 22 in stage IIA, and 18 in stage IIB. The cases were also divided into squamous cell carcinoma (51 cases), adenocarcinoma (6 cases), and other (2 cases) according to the pathological type. The degree of tumor differentiation was divided into well differentiated (12 cases), moderately differentiated (22 cases), and poorly differentiated (25 cases). In terms of infiltration depth, 27 cases were cervix < 1/3 deep, 10 cases were 1/3 to 2/3 deep, and 22 cases were >2/3 deep. Regarding tumor size, 44 cases were ≤4 cm and 15 cases were >4 cm (Table 1).

### 3.2. Analysis of the SLN Examination

An SLN was detected in 56 of 59 patients with cervical cancer, with a detection rate of 94.92%. Histopathological examination confirmed the presence of lymph node metastasis in 15 of 59 patients (SLN metastasis, 13; NSLN metastasis, 2), with a metastasis rate of 25.42% (15/59). The calculated sensitivity of SLNB was 86.67% (13/15), and the false-negative rate was 13.33% (2/15).

### 3.3. Analysis of SLN Examination in Patients with Cervical Cancer with Different Pathological Features

Statistical analysis revealed that there was no significant difference in the positive rate of SLN among patients with cervical cancer with different FIGO stages (stage I, 32.14%; stage IIa, 35.71%; and stage IIb, 32.14%), pathological types (squamous cell carcinoma, 87.50%; adenocarcinoma, 8.93%; and others, 3.57%), differentiation degree (high differentiation, 19.64%; medium differentiation, 37.50%; and low differentiation, 42.86%), infiltration depth (<1/3, 42.86%; 1/3-2/3, 17.86%; and >2/3, 39.29%),and tumor size (≤4 cm, 73.21%; >4 cm, 26.79%) (*P* > 0.05) (Table 1).

### 3.4. Correlation Analysis between the Positive Rate of SLN Detection and Pathological Characteristics of Cervical Cancer

The Pearson correlation test showed that the positive rate of SLN detection had no significant correlation with FIGO staging, pathological type, differentiation degree, infiltration depth, and tumor size of cervical cancer (*P* > 0.05) (Table 2).

## 4. Discussion

Cervical cancer is the second most common malignancy in developing countries. In recent years, with the popularization of screening range and the continuous improvement of screening methods, the incidence of cervical cancer in the younger population is on the rise [10]. A survey showed that the main treatment measures for early cervical cancer were extensive hysterectomy, double adnexectomy, and pelvic lymph node dissection, with or without abdominal para-aortic lymph node dissection. However, most patients do not need a lymph node dissection. With the improvement of treatment strategies, the postoperative survival of cervical cancer patients has been significantly prolonged. However, some patients may develop immune dysfunction, lower extremity edema, and other related complications after lymph node removal, which affects the prognosis [11, 12]. Therefore, an accurate assessment of cervical cancer lymph node metastasis and pathological features is of great significance.

MRI, CT, ultrasound, and positron emission tomography (PET) are important imaging techniques for the clinical evaluation of lymph node metastasis, mainly by measuring the lymph node volume to assess the presence of lymph node metastasis. However, clinical findings show that some patients do not have lymphadenopathy despite the presence of lymph node metastasis, which leads to a less accurate diagnosis [13]. Although PET-CT can accurately assess distant metastasis, the cost is relatively high [14]. In addition, the role of SLN in primary tumor lymph node metastasis is very important. Therefore, SLN metastases can indicate the presence of lymph node metastases in this region, thus requiring lymph node dissection. However, if no SLN metastases have occurred, unnecessary lymph node dissection can be avoided. Based on this background, SLNB has been widely used in breast cancer and plays an important role in melanoma surgery. To avoid the deficiency of extensive hysterectomy combined with lymph node dissection in the treatment of early cervical cancer, SLNB provides a new idea and method for the preoperative evaluation of lymph node metastasis and invasion in patients with cervical cancer. Moreover, the regularity of lymphatic drainage in patients with cervical cancer and main transfer path of the disease (lymphatic metastasis) provides a theoretical feasibility of SLNB in the diagnosis and treatment of cervical cancer. Relevant studies abroad have confirmed that the detection rate of SLN in cervical cancer is as high as 90%-100%, that the sensitivity can reach 80%-100%, and that the false-negative rate is less than 10% [15, 16]. Our results showed that the detection rate of SLN in 59 cervical cancer patients was 94.92%, the rate of lymph node metastasis was 25.42%, the sensitivity of SLNB was 86.67%, and the false-negative rate was 13.33%, which was consistent with the results of previous studies, indicating that SLNB is in the cervical It has high application value and high sensitivity in the diagnosis and evaluation of cancer lymph node metastasis.

In addition, Cusimano et al. [17] and Balaya et al. [18] showed that the influencing factors of SLN detection mainly included the clinicopathological features of patients with cervical cancer, such as preoperative cervical conization, preoperative radiotherapy and chemotherapy, local tumor size, FIGO stage, and others. In addition, the combined method was used to perform SLN examination for locally advanced and early cervical cancer. It was found that there were significant differences in the SLN detection rates among patients with different stages, and they were closely related to tumor size. There was no significant difference in the SLN examination among patients with cervical cancer with different pathological characteristics (FIGO stage, pathological type, differentiation degree, infiltration depth, and tumor size). The Pearson correlation test showed no significant correlation between the positive rate of SLN detection and FIGO stage, pathological type, differentiation degree, infiltration depth, and tumor size of cervical cancer (*P* > 0.05), indicating that there was no correlation between the pathological characteristics of cervical cancer and SLNB examination. Therefore, the disease stage, degree of differentiation, and infiltration depth of the tumor did not affect the results of SLNB. There was a certain difference with the above research, which may be related to the different range of case selection. A large number of foreign studies confirmed that SLNB combined with extensive cervical resection can effectively reduce intraoperative blood loss, shorten the operation time, and reduce the risk of complications, which is significant to improve the quality of life of patients [19, 20]. Therefore, SLNB is safe, is feasible, and has a high application value in the early diagnosis and evaluation of cervical cancer lymph node metastasis.

## 5. Conclusion

In conclusion, SLNB has high sensitivity, safety, and feasibility in the diagnosis and evaluation of cervical cancer lymph node metastasis, and SLNB examination has no significant correlation with the clinicopathological characteristics of cervical cancer. However, this study is a single-center study with a small sample size, and there are few systematic studies on the correlation between SLNB and the pathological characteristics of cervical cancer. Therefore, it is still necessary to expand the scope of sample selection and increase the sample size in the future to further confirm the conclusions of this study.

## Figures and Tables

**Table 1:** Analysis of SLN examination in patients with cervical cancer with different pathological features.

Pathological features		Number of samples	Positive	Negative	*χ* ^2^ value	*P* value
FIGO staging	Stage I	19	18 (32.14)	1 (33.33)	1.697	0.428
	Stage IIa	22	20 (35.71)	2 (66.67)		
	Stage IIb	18	18 (32.14)	0 (0.00)		

Pathological type	Squamous cell carcinoma	51	49 (87.50)	2 (66.67)	1.918	0.383
	Adenocarcinoma	6	5 (8.93)	1 (33.33)		
	Other	2	2 (3.57)	0 (0.00)		

Differentiation degree	High differentiation	12	11 (19.64)	1 (33.33)	0.337	0.845
	Medium differentiation	22	21 (37.50)	1 (33.33)		
	Low differentiation	25	24 (42.86)	1 (33.33)		

Infiltrative depth	<1/3	27	24 (42.86)	3 (100.00)	3.746	0.154
	1/3~2/3	10	10 (17.86)	0 (0.00)		
	>2/3	22	22 (39.29)	0 (0.00)		

Tumor size	≤4 cm	44	41 (73.21)	3 (100.00)	0.128	0.721
	>4 cm	15	15 (26.79)	0 (0.00)		

**Table 2:** Correlation analysis between the positive rate of SLN detection and pathological characteristics of cervical cancer.

Items		FIGO staging	Pathological type	Differentiation degree	Infiltrative depth	Tumor size
Positive rate of SLN detection	*r* value	0.213	0.322	0.197	0.206	0.182
	*P* value	0.813	0.544	1.212	0.892	0.981

## Data Availability

The data will be available upon reasonable requests from the corresponding author.
